# Textbook, multimedia, and generative AI in elementary English learning: usage patterns and family-reported evaluations

**DOI:** 10.3389/fpsyg.2026.1870251

**Published:** 2026-10-07

**Authors:** Lu Sun, XiaCheng Song, Jing Jin, Min Dai, Huafeng Qu, Xiqiong Yi, Huirong Huang

**Affiliations:** 1 Xi'an Fanyi University, Xi'An, China; 2 Guangdong University of Science and Technology, Dongguan, China; 3 Taizhou University, Taizhou, China; 4 Yunnan College of Business Management, Kunming, China; 5 Dongguan Polytechnic, Dongguan, China; 6 Guangdong Industry Polytechnic University, Guangzhou, China

**Keywords:** educational technology, elementary English learning, family-reported evaluation, generative AI, learning modality

## Abstract

**Introduction:**

This study examined how often elementary-school learners used textbooks, multimedia resources, and generative AI tools and how these modalities were evaluated by family respondents.

**Methods:**

A non-probability, teacher-mediated online survey yielded 987 consented responses from multiple sites in mainland China. Respondents rated usage frequency and 15 evaluation items crossing five dimensions with three modalities.

**Results:**

Textbooks were used most frequently and received the most favorable ratings. After applying a single-step multivariate-t adjustment to each family of three within-dimension contrasts, multimedia and AI differed reliably only for enjoyment in the full sample. The original three-modality confirmatory model had poor absolute fit (RMSEA = 0.151). A crossed dimension-by-modality model improved fit (RMSEA = 0.067) but produced a negative residual variance and was therefore inadmissible; latent achievement regression was not retained. In the primary proportional-odds model using observed modality scores and categorical controls, textbook evaluation was positively associated with family-reported achievement band (*b* = 0.343, OR = 1.409, *p* < 0.001), whereas multimedia (*p* = 0.085) and AI (*p* = 0.808) were not.

**Discussion:**

These cross-sectional associations describe this teacher-mediated convenience sample and do not establish modality effects on achievement.

## 1 Introduction

Generative AI is now readily accessible for language practice, feedback, and interaction, but availability alone does not establish educational advantage. Elementary learners encounter textbooks, conventional multimedia resources, and AI tools within the same learning ecology. These resources differ in curricular structure, adult oversight, interactivity, feedback, and novelty, so comparisons should not collapse them into a simple print-vs.-digital contrast. Research on generative AI in education describes both opportunities for adaptive feedback and risks of shallow engagement, dependence, and uneven instructional integration (Cosentino et al., 2025; Fan et al., 2025; Mittal et al., 2024; Yan et al., 2025). In language learning specifically, observed use varies by task, duration, perceived usefulness, and the learner's ability to negotiate tool affordances and constraints (Hwang et al., 2025; Lee et al., 2026; Liu et al., 2025). These findings make reported exposure and instructional context essential when interpreting favorable or unfavorable evaluations of AI. This comparative problem is especially important in elementary education, where resource choice is embedded in adult decisions and school routines. Textbooks generally provide a stable curricular sequence and a visible record of assigned work; multimedia resources combine audiovisual input with structured practice; and generative AI permits open-ended interaction whose quality depends heavily on prompts, verification, and supervision. A family rating can therefore reflect usability, familiarity, confidence, or alignment with schoolwork as well as perceived learning value. Treating the three modalities within one design makes these trade-offs visible while avoiding the assumption that novelty and educational effectiveness are interchangeable.

A second issue concerns what modality evaluations measure. Studies of language-learning enjoyment, AI-assisted speaking, ChatGPT use, and readiness show that effectiveness, ease, enjoyment, trust, and continuance can be related but non-equivalent aspects of technology experience (Botes et al., 2021; Huang and Zou, 2024; Luo and Zou, 2025; Nemt-allah et al., 2024). When the same prompts are repeated for several resources, however, responses can reflect both the intended dimensions and shared reactions to the resource named in each block. High internal consistency within modality may consequently represent general favourability or halo responding rather than five distinct constructs. Educational-measurement guidance therefore emphasizes construct validity, absolute fit, discriminability, residual dependencies, and admissibility rather than selecting a model from relative fit alone (Chang, 2024; Goretzko et al., 2024; Kaldaras et al., 2024; McNeish and Wolf, 2023). Validity concerns the defensibility of an intended score interpretation; it is not established by coefficient alpha or by a model fitting better than weaker alternatives. This distinction is central here because a poorly fitting measurement model cannot support strong latent-predictor claims.

Against this background, the study compares family-reported usage and evaluations of textbooks, multimedia resources, and generative AI tools in elementary English learning. Research comparing paper and screen reading indicates that medium-related differences depend on task and context rather than a universal digital advantage (Clinton, 2019; Delgado et al., 2018; Mangen and van der Weel, 2016). Work on human-AI complementarity similarly argues that educational value depends on how technology is coupled with human guidance and established learning routines (Holstein and Aleven, 2022; Kern, 2024). We therefore establish a behavioral baseline, compare five evaluation prompts across modalities, and examine competing measurement representations in independent exploratory and confirmatory subsamples. Achievement is treated as an ordered family-reported band and analyzed with transparent observed modality scores after the latent structure failed to provide a defensible basis for structural regression. Because recruitment occurred through teachers' class messaging groups and the export did not retain school, class, or respondent-type identifiers, the study is framed as a non-probability, teacher-mediated, multi-site family survey rather than a nationally representative assessment.

**Research questions**

**RQ1**. How do reported usage frequencies differ across textbook, multimedia, and AI-based English-learning modalities among elementary-school learners?

**RQ2**. How do family-reported evaluations differ across modalities and across five dimensions: effectiveness, ease of use, enjoyment, concern, and continuance intention?

**RQ3**. How well do modality-based, dimension-based, and crossed dimension-by-modality representations account for the evaluation items?

**RQ4**. Are observed modality-evaluation scores associated with family-reported English achievement band after adjustment for measured background characteristics?

## 2 Methods

### 2.1 Study design and data-collection procedure

This study used a cross-sectional, non-probability online survey administered to families of elementary-school students at multiple sites in mainland China (Cornesse et al., 2020). Homeroom teachers distributed the questionnaire through class messaging groups. Data were collected from September 20 to September 27, 2025. The exported dataset did not include school identifiers, class identifiers, or a variable distinguishing parent completion from student completion with parental assistance. Accordingly, school/class clustering and respondent-type comparisons could not be estimated, and all reports are described as family responses rather than verified parent-only reports.

### 2.2 Participants and analytic sample

The exported dataset contained 1,003 complete rows. Sixteen respondents did not provide consent, leaving an analytic sample of *N* = 987. The survey platform did not retain the number of partial attempts excluded before export. The sample was concentrated in Grade 5 and county/township locations and should not be interpreted as representative of families across mainland China.

### 2.3 Measures

Modality usage frequency was assessed with Q16 for textbooks, multimedia courses/apps, and AI tools using five anchors: 1 = never, 2 = rarely (a few times per month), 3 = sometimes (1–2 times per week), 4 = often (3–4 times per week), and 5 = almost every day. Evaluations comprised 15 ordered-category items formed by crossing five prompts—perceived effectiveness, ease of use, enjoyment, concern, and continuance intention—with the three modalities. Higher values indicated more favorable evaluations; for concern, higher values indicated less concern. Modality scores used in the observed-score achievement model were the arithmetic means of the five consistently oriented items within each modality. Achievement was reported in five ordered bands based on the child's previous-semester English final examination: 59 or below, 60–79, 80–90, 90–95, and above 95. The adjacent bands overlapped at 90 and 95 in the administered questionnaire, and examinations were set locally rather than standardized across grades or schools. We retained the administered categories rather than reconstructing unavailable raw scores and treated the outcome as ordinal. Full Q16-Q21 wording and response anchors are reproduced in Supplementary Table 7.

### 2.4 Data quality control and preprocessing

The survey platform restricted repeated submissions from the same IP address. The analytic sample was defined by consent (Q1 = 1). The exported data contained no item-level missing values, but the platform did not provide a count of incomplete attempts removed before export. Using seed 20251214, the consented sample was randomly divided into independent EFA (*n* = 493) and CFA (*n* = 494) subsamples. Item descriptive statistics and internal-consistency estimates are reported in Supplementary Table 1, and full item wording and response anchors are reported in Supplementary Table 7.

### 2.5 Statistical analysis

Reported usage and evaluation ratings were compared with linear mixed-effects models containing participant-specific random intercepts for the within-person repeated measures (Bates et al., 2015). For Q16 usage, the three pairwise modality contrasts formed one multiplicity family. Holm adjustment was applied to the *p* values; because Holm-adjusted confidence limits are not defined in emmeans, simultaneous 95% confidence intervals used the Bonferroni adjustment over the same three contrasts (Lenth and Piaskowski, 2026). For each evaluation dimension, three modality contrasts constituted one multiplicity family; *p* values and simultaneous 95% confidence intervals used the same single-step multivariate-t adjustment implemented in emmeans. Repeated-measures Cohen's d (d_z) described paired effect sizes. School- and class-level random effects could not be fitted because those identifiers were not retained.

Psychometric evidence was evaluated in the split samples. EFA used polychoric correlations, principal-axis extraction, oblimin rotation, and parallel analysis (Supplementary Table 2). The weak fourth EFA factor accounted for only 4.6% of variance and consisted mainly of secondary concern-item loadings, rather than a coherent substantive factor, so it was not carried forward as a confirmatory candidate. CFA used WLSMV for ordered indicators in (Rosseel 2012). In addition to the original Book-vs.-Digital, three-modality, and five-dimension models, we tested a three-modality model with correlations among same-dimension residuals and a crossed correlated-trait correlated-method specification containing dimension and modality factors simultaneously. Convergence, absolute fit, negative residual variances, and the positive definiteness of latent covariance matrices were evaluated jointly; an inadmissible solution was not interpreted even when global fit indices appeared favorable. Original three-modality loadings and latent correlations are retained as diagnostics in Supplementary Table 3. Because no admissible latent structure provided sufficiently strong support for the original latent achievement regression, the primary achievement analysis used observed mean modality scores in a cumulative-logit proportional-odds model (Christensen, 2025). Grade, gender, region, parental education, extracurricular-learning time, and screen time were entered categorically; interest and binary home-resource indicators were included as recorded. The complete coefficient table and nominal-effect tests are reported in Supplementary Table 4. Sensitivity analyses restricted the sample to families reporting at least weekly use of both multimedia and AI (Supplementary Table 5), standardized achievement within grade (Supplementary Table 6), and fitted five separate proportional-odds models in which the textbook, multimedia, and AI ratings for one evaluation dimension were entered together (Supplementary Table 8). For the dimension-level models, Holm adjustment was applied across the five dimension-specific tests within each modality. Analyses were run in R 4.6.0 with lavaan 0.6–21, ordinal 2025.12–29, emmeans 2.0.3, and lme4 2.0-1. During preparation of this manuscript, the authors used OpenAI Codex (GPT-5; OpenAI, https://openai.com) for language editing, statistical-code review, and internal consistency checking. The tool did not generate the data, execute the statistical analyses, or determine the interpretation of results. The authors executed all analyses and critically reviewed and verified all code, outputs, and interpretations. The authors take full responsibility for the accuracy, originality, and integrity of the work.

### 2.6 Ethics

The study protocol was approved by the Research Ethics Committee of Xi'an Fanyi University (XFU-REC; Ref. No. XFU-REC/2025/09/1801; approved on September 18, 2025; approval period: September 20, 2025 to December 25, 2025). Written parental/guardian informed consent and child assent were obtained electronically from September 20 to September 27, 2025 (the full data-collection period), after ethical approval had been granted. Informed consent was obtained electronically from a parent or legal guardian prior to participation, and child assent was also obtained where applicable. Participation was voluntary, and all data were collected and managed in accordance with the approved protocol with confidentiality safeguards.

## 3 Results

### 3.1 Sample characteristics and analytic split

The analytic sample included 987 consented family responses and was randomly split into EFA (*n* = 493) and CFA (*n* = 494) subsamples. Grade 5 accounted for 43.9% of cases, whereas Grade 6 accounted for 2.4%. Gender was nearly balanced. Most respondents were located in county/township areas (73.4%), with 25.6% in urban areas and 1.0% in rural areas. The most common highest parental education category was undergraduate education (28.2%). These distributions reinforce the non-probability, non-national scope of the sample (Table 1).

**Table 1:** Sample characteristics for the full analytic sample and EFA/CFA split.

Characteristic	Overall (*N* = 987)	EFA (*N* = 493)	CFA (*N* = 494)
Grade			
G1	90 (9.1%)	46 (9.3%)	44 (8.9%)
G2	60 (6.1%)	31 (6.3%)	29 (5.9%)
G3	177 (17.9%)	91 (18.5%)	86 (17.4%)
G4	203 (20.6%)	94 (19.1%)	109 (22.1%)
G5	433 (43.9%)	219 (44.4%)	214 (43.3%)
G6	24 (2.4%)	12 (2.4%)	12 (2.4%)
Gender			
Male	501 (50.8%)	251 (50.9%)	250 (50.6%)
Female	486 (49.2%)	242 (49.1%)	244 (49.4%)
Only-child status: yes	357 (36.2%)	180 (36.5%)	177 (35.8%)
Region			
Urban	253 (25.6%)	133 (27.0%)	120 (24.3%)
County/township	724 (73.4%)	356 (72.2%)	368 (74.5%)
Rural	10 (1.0%)	4 (0.8%)	6 (1.2%)
Mother at home			
Mostly at home	850 (86.1%)	424 (86.0%)	426 (86.2%)
Partly at home	108 (10.9%)	52 (10.5%)	56 (11.3%)
Mostly away	29 (2.9%)	17 (3.4%)	12 (2.4%)
Father at home			
Mostly at home	504 (51.1%)	248 (50.3%)	256 (51.8%)
Partly at home	338 (34.2%)	176 (35.7%)	162 (32.8%)
Mostly away	145 (14.7%)	69 (14.0%)	76 (15.4%)
Highest parental education			
Junior secondary or below	166 (16.8%)	82 (16.6%)	84 (17.0%)
Senior secondary	265 (26.8%)	127 (25.8%)	138 (27.9%)
College	229 (23.2%)	124 (25.2%)	105 (21.3%)
Undergraduate	278 (28.2%)	137 (27.8%)	141 (28.5%)
Postgraduate	49 (5.0%)	23 (4.7%)	26 (5.3%)
Home English-learning resources			
English books/picture books	400 (40.5%)	190 (38.5%)	210 (42.5%)
English-learning app/platform	285 (28.9%)	144 (29.2%)	141 (28.5%)
Parent can use English with child	127 (12.9%)	56 (11.4%)	71 (14.4%)
Quiet study place	676 (68.5%)	327 (66.3%)	349 (70.6%)
No listed home resource	135 (13.7%)	74 (15.0%)	61 (12.3%)
English-learning interest			
Very low	23 (2.3%)	11 (2.2%)	12 (2.4%)
Low	44 (4.5%)	29 (5.9%)	15 (3.0%)
Moderate	311 (31.5%)	151 (30.6%)	160 (32.4%)
High	316 (32.0%)	157 (31.8%)	159 (32.2%)
Very high	293 (29.7%)	145 (29.4%)	148 (30.0%)
Extracurricular English-learning time			
Almost none	270 (27.4%)	144 (29.2%)	126 (25.5%)
About 1 h	290 (29.4%)	137 (27.8%)	153 (31.0%)
1–2 h	242 (24.5%)	126 (25.6%)	116 (23.5%)
2–3 h	113 (11.4%)	52 (10.5%)	61 (12.3%)
More than 4 h	72 (7.3%)	34 (6.9%)	38 (7.7%)
Extracurricular English-learning type			
None	430 (43.6%)	224 (45.4%)	206 (41.7%)
Occasional courses	179 (18.1%)	88 (17.8%)	91 (18.4%)
Weekly online courses	78 (7.9%)	36 (7.3%)	42 (8.5%)
Weekly offline courses	263 (26.6%)	125 (25.4%)	138 (27.9%)
Weekly online and offline courses	37 (3.7%)	20 (4.1%)	17 (3.4%)
Daily screen time			
< 1 hour	607 (61.5%)	310 (62.9%)	297 (60.1%)
1–2 h	320 (32.4%)	149 (30.2%)	171 (34.6%)
3–4 h	41 (4.2%)	23 (4.7%)	18 (3.6%)
5 h	19 (1.9%)	11 (2.2%)	8 (1.6%)
Start of systematic English learning			
Preschool	127 (12.9%)	62 (12.6%)	65 (13.2%)
G1–G2	278 (28.2%)	141 (28.6%)	137 (27.7%)
G3–G4	561 (56.8%)	281 (57.0%)	280 (56.7%)
G5–G6	21 (2.1%)	9 (1.8%)	12 (2.4%)
Family-reported English achievement band			
59	69 (7.0%)	44 (8.9%)	25 (5.1%)
60–79	128 (13.0%)	68 (13.8%)	60 (12.1%)
80–90	246 (24.9%)	120 (24.3%)	126 (25.5%)
90–95	296 (30.0%)	144 (29.2%)	152 (30.8%)
95+	248 (25.1%)	117 (23.7%)	131 (26.5%)

Values are *n* (%). Percentages are column percentages. The analytic sample included respondents who provided informed consent. The EFA/CFA split was generated by random assignment after eligibility filtering. Multiple selections were permitted for home English-learning resources; percentages for those items therefore do not sum to 100%.

### 3.2 Descriptive profiles and modality usage baseline

Modality usage differed substantially. Textbook usage was highest (*M* = 3.83, SD = 1.31), followed by AI (*M* = 2.49, SD = 1.31) and multimedia (*M* = 2.28, SD = 1.28). The modality omnibus test was significant, Wald chi-square (2) = 1141.0, *p* < 0.001, and all Holm-adjusted usage contrasts were significant (Table 2).

Table 2Reported usage frequency across learning modalities.

**Table:** 

Panel A. Descriptive statistics				
**Modality**	***N***	**Mean**	**SD**	**Median**
Textbook	987	3.83	1.31	4
Multimedia	987	2.28	1.28	2
AI	987	2.49	1.31	2

**Table:** 

**Panel B. Omnibus modality effect**			
**Term**	**Wald** χ^2^	**df**	***p***
Modality	1,141.00	2	< 0.001

**Table:** 

**Panel C. Pairwise modality contrasts**				
**Contrast**	**Estimate**	**95% CI**	**p_adj**	**d_z**
Textbook—Multimedia	1.55	[1.43, 1.67]	< 0.001	0.91
Textbook—AI	1.34	[1.22, 1.46]	< 0.001	0.80
Multimedia—AI	−0.21	[–0.33, −0.09]	< 0.001	−0.16

Usage frequency was rated from 1 to 5, with higher scores indicating more frequent use. Pairwise contrasts are estimated marginal mean differences from a linear mixed-effects model with participant-specific random intercepts. Across the family of three contrasts, p_adj values were Holm-adjusted and simultaneous 95% confidence intervals were Bonferroni-adjusted. d_z denotes repeated-measures Cohen's d.

Low exposure was common: 38.3% reported never using multimedia and 58.7% reported never or rarely using it; the corresponding proportions for AI were 30.4% and 53.9% (Supplementary Table 5, Panel A).

Estimated marginal means showed that textbooks received the highest ratings across all five dimensions. Multimedia and AI clustered more closely. Their full-sample difference was reliable for enjoyment, but not for effectiveness, ease, concern, or continuance intention after coherent multiplicity adjustment (Figure 1; Table 3).

**Figure 1:**
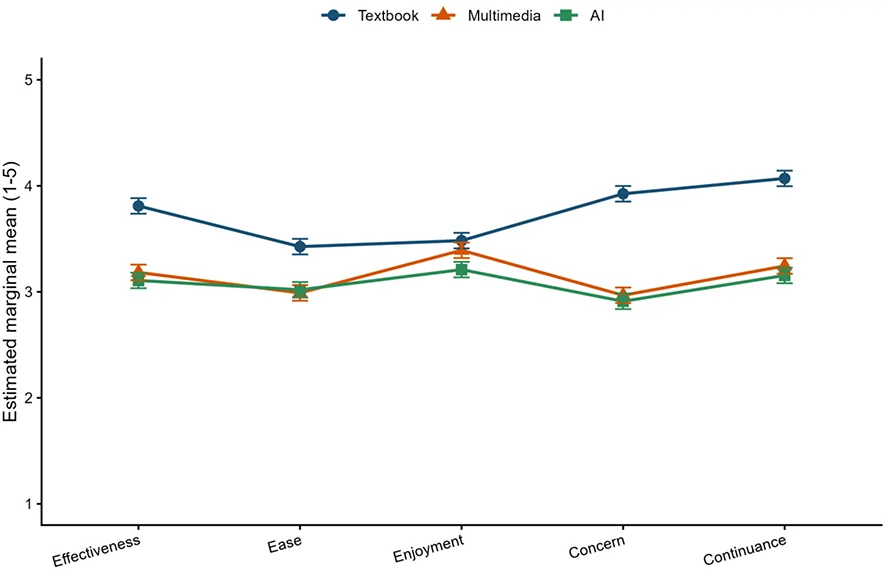
Estimated marginal means of family-reported evaluations across five dimensions by learning modality. Points indicate estimated marginal means and error bars indicate 95% confidence intervals. The full administered response range (1–5) is displayed. Higher scores indicate more favorable evaluations; for concern, higher scores indicate lower concern.

Table 3Dimension-wise modality effects and pairwise contrasts.

**Table:** 

**Panel A. Omnibus modality effects by evaluation dimension**			
**Dimension**	**Wald** χ^2^	**Df**	***p***
Effectiveness	324.41	2	< 0.001
Ease of use	150.44	2	< 0.001
Enjoyment	45.39	2	< 0.001
Concern	844.97	2	< 0.001
Continuance intention	675.92	2	< 0.001

**Table:** 

**Panel B. Pairwise modality contrasts within each dimension**					
**Dimension**	**Contrast**	**Estimate**	**Simultaneous 95% CI**	**p_adj**	**d_z**
Effectiveness	Textbook–Multimedia	0.627	[0.527, 0.728]	< 0.001	0.430
Effectiveness	Textbook–AI	0.703	[0.603, 0.804]	< 0.001	0.493
Effectiveness	Multimedia–AI	0.076	[−0.025, 0.177]	0.179	0.067
Ease of use	Textbook–Multimedia	0.437	[0.343, 0.530]	< 0.001	0.334
Ease of use	Textbook–AI	0.407	[0.314, 0.501]	< 0.001	0.294
Ease of use	Multimedia–AI	−0.029	[−0.123, 0.064]	0.741	−0.029
Enjoyment	Textbook–Multimedia	0.091	[−0.006, 0.188]	0.071	0.066
Enjoyment	Textbook–AI	0.274	[0.177, 0.371]	< 0.001	0.196
Enjoyment	Multimedia–AI	0.182	[0.085, 0.279]	< 0.001	0.164
Concern	Textbook–Multimedia	0.957	[0.865, 1.049]	< 0.001	0.712
Concern	Textbook–AI	1.014	[0.922, 1.106]	< 0.001	0.726
Concern	Multimedia–AI	0.057	[−0.035, 0.149]	0.317	0.064
Continuance intention	Textbook–Multimedia	0.826	[0.735, 0.917]	< 0.001	0.632
Continuance intention	Textbook–AI	0.915	[0.824, 1.006]	< 0.001	0.676
Continuance intention	Multimedia–AI	0.089	[−0.002, 0.180]	0.057	0.093

Each dimension was analyzed using a linear mixed-effects model with modality as a fixed effect and participant-specific random intercepts. Within each dimension, the three pairwise contrasts formed one multiplicity family. p_adj values and simultaneous 95% confidence intervals used a single-step multivariate-t adjustment. d_z denotes repeated-measures Cohen's d. Higher scores indicate more favorable evaluations; for concern, higher scores indicate lower concern.

Dimension-level comparisons showed that textbooks were rated more favorably than both digital modalities across all five dimensions. Multimedia and AI did not differ for effectiveness, ease, concern, or continuance intention after coherent multiplicity adjustment. Multimedia received a higher enjoyment rating than AI [difference = 0.182, simultaneous 95% CI (0.085, 0.279), *p* < 0.001]. The textbook-multimedia enjoyment contrast (*p* = 0.071) and multimedia-AI continuance contrast (*p* = 0.057) were not statistically reliable (Table 3).

### 3.3 Competing CFA models and measurement quality (CFA; WLSMV)

The original three-modality model had the best relative fit among the three initially specified models but poor absolute fit, chi-square (87) = 1066.84, CFI = 0.926, TLI = 0.911, RMSEA = 0.151, SRMR = 0.070. Adding correlations among residuals that shared the same evaluation dimension improved fit (CFI = 0.979, RMSEA = 0.089, SRMR = 0.039) and produced an admissible solution. The fully crossed dimension-by-modality CT-CM model improved global fit further (CFI = 0.990, RMSEA = 0.067, SRMR = 0.030) but produced a negative residual variance and failed the model post-check. It was therefore inadmissible and was not used for substantive inference (Table 4).

**Table 4:** Confirmatory factor analysis model comparison.

**Model**	Description	Scaled chi-square	df	CFI	TLI	RMSEA	SRMR	Admissibility
1	Book vs. Digital	1,133.88	89	0.921	0.907	0.154	0.085	Admissible; poor fit
2	Three-modality	1,066.84	87	0.926	0.911	0.151	0.070	Admissible; poor fit
3	Five dimension	3,093.88	80	0.773	0.702	0.276	0.200	Inadmissible latent covariance
4	Three-modality + same-dimension residual covariances	353.05	72	0.979	0.969	0.089	0.039	Admissible; residual misfit remains
5	Crossed dimension-by-modality CT-CM	200.25	62	0.990	0.982	0.067	0.030	Inadmissible negative residual variance

Models were estimated in the CFA subsample (*n* = 494) using WLSMV for ordered indicators. Fit indices were considered together with admissibility diagnostics. The crossed CT-CM solution was not interpreted because it contained a negative residual variance. The correlated-residual model improved fit but still indicated non-negligible misspecification.

### 3.4 Measurement structure determination

EFA reproduced a strong modality-blocked loading pattern, with parallel analysis suggesting a weak fourth factor related mainly to concern items (Supplementary Table 2). That fourth factor explained only 4.6% of variance and was defined by secondary loadings from two concern items rather than a coherent construct; it was therefore not advanced to CFA. The crossed CFA demonstrated that better global fit could be obtained only with an inadmissible solution, while the original modality factors also left substantial same-dimension residual dependence. High within-modality alpha values (0.869–0.908; Supplementary Table 1) and a Multimedia-AI latent correlation of 0.847 (Supplementary Table 3) further show that the instrument cannot distinguish modality-specific psychological organization from general favorability, block presentation, and shared method variance. We therefore treat the measurement analyses as a diagnostic description of the instrument rather than validation of three separable latent predictors.

### 3.5 Observed-score associations with family-reported English achievement band

The primary achievement analysis used a proportional-odds model with observed modality-evaluation scores and measured background controls. Textbook evaluation was positively associated with a higher family-reported achievement band ([*b* = 0.343, 95% CI (0.182,0.503), OR = 1.409, *p* < 0.001]. Multimedia (*b* = 0.147, *p* = 0.085) and AI (unrounded *b* = −0.01968, OR = 0.981, *p* = 0.808) were not significant, and their coefficients did not differ in the full sample (*p* = 0.268). Odds ratios were calculated from unrounded coefficients. Nominal-effect tests did not detect a proportional-odds violation at *p* < 0.05, although region was borderline (*p* = 0.051). The full model and diagnostics appear in Supplementary Table 4; the three focal coefficients are summarized in Table 5.

**Table 5:** Controlled proportional-odds associations with family-reported English achievement band.

Predictor	*b* (OR)	95% CI for b	*p*
Textbook evaluation score	0.343 (1.409)	[0.182, 0.503]	< 0.001
Multimedia evaluation score	0.147 (1.158)	[−0.020, 0.313]	0.085
AI evaluation score	−0.020 (0.981)	[−0.179, 0.139]	0.808

Coefficients are cumulative-logit estimates from the full-sample proportional-odds model (*N* = 987). Odds ratios above 1 indicate greater odds of being in a higher reported achievement band and were calculated from unrounded coefficients. The model adjusted for grade, gender, region, parental education, English-learning interest, extracurricular English-learning time, screen time, books, learning-app access, and a quiet study place. Associations are cross-sectional and non-causal. Three home-resource indicators were included as covariates; parental ability to use English with the child, described in Table 1, was not included in this model. The confidence intervals are for b, not for the odds ratios.

Sensitivity results were not uniform. After standardizing achievement within grade, textbook remained positively associated with the outcome, multimedia was small and borderline, and AI remained non-significant (Supplementary Table 6). Among the 293 respondents who reported using both multimedia and AI at least weekly, multimedia was positively associated with achievement band (*b* = 0.654, *p* = 0.008), AI was not (*b* = −0.273, *p* = 0.241), and their coefficients differed (*p* = 0.035; Supplementary Table 5, Panel B). This subgroup represented 29.7% of the analytic sample, was selected on exposure, and followed a non-significant full-sample contrast among several sensitivity analyses. Its *p* values were not adjusted across that analysis sequence, and the model was especially vulnerable to the unavailable school/class clustering. It is therefore reported as exploratory rather than as a replacement for the full-sample model. In five dimension-specific models, the textbook coefficients remained positive after Holm adjustment for effectiveness (*b* = 0.208, p_adj = 0.003), ease (*b* = 0.350, p_adj < 0.001), and enjoyment (*b* = 0.213, p_adj = 0.005), but not for lower concern (*b* = 0.104, p_adj = 0.145) or continuance intention (*b* = 0.115, p_adj = 0.145). Thus, the composite textbook association was not carried primarily by the low-concern item (Supplementary Table 8).

## 4 Discussion

### 4.1 Principal findings and overall empirical pattern

Across this non-probability, teacher-mediated sample, textbooks were reported as both the most frequently used and the most positively evaluated modality. Multimedia and AI were used much less often, and more than half of respondents reported never or rarely using each digital modality. These descriptive results matter because ratings from non-users or rare users can reflect expectations and prior attitudes rather than direct learning experience. The combination of high textbook exposure and favorable textbook ratings is compatible with familiarity, curricular alignment, and repeated opportunities to observe outcomes; the survey cannot separate those mechanisms. Conversely, lower digital ratings should not be read as direct evidence that the resources are intrinsically less effective, because many respondents had little exposure on which to base an evaluation. The full-sample proportional-odds model showed an association between textbook evaluation and family-reported achievement band, whereas multimedia and AI were not significant. This pattern does not establish that textbooks improve achievement. It describes covariation within a convenience sample recruited through schools and measured with locally defined examinations. Evidence on AI adoption and trust likewise cautions that favorable ratings depend on perceived usefulness, social reinforcement, transparency, and context rather than technical capability alone (Glikson and Woolley, 2020; Shata and Hartley, 2025; Shin, 2021).

### 4.2 Measurement structure, method variance, and model limitations

The measurement results do not justify a claim that families possess distinct modality-based psychological structures. The original modality CFA fit poorly in absolute terms, and Multimedia and AI were highly correlated. Modeling same-dimension residual dependencies improved fit, while a fully crossed trait-method model produced an inadmissible negative variance. The modality blocks may therefore have encouraged internally consistent responses to each named resource, and the high alpha values may reflect a general evaluative halo. The diagnostic models also show why the original three-factor solution should not be rescued solely because its relative fit exceeded the Book-vs-Digital and five-dimension alternatives: all three initial specifications left substantial unexplained covariance, and the most flexible crossed model failed an admissibility check. At the same time, the observed mean differences remain descriptively useful because they summarize how the modalities were rated without treating those summaries as validated latent traits. This restrained interpretation follows the principle that construct meaning cannot be inferred from relative model fit when absolute fit and discriminability remain inadequate (Chang, 2024; Goretzko et al., 2024; McNeish and Wolf, 2023).

### 4.3 Limited and exposure-dependent differences between multimedia and AI

Full-sample pairwise contrasts provided little evidence that multimedia and AI were consistently distinguished. After aligning adjusted *p* values with simultaneous intervals, the two modalities differed reliably only for enjoyment; the continuance contrast was not significant. A different pattern appeared in the smaller weekly-exposure subgroup, where multimedia was positively associated with achievement and differed from AI (Supplementary Table 5, Panel B). This exposure-conditioned result may reflect actual experience, but it followed a non-significant full-sample contrast, was one of several sensitivity analyses, and was not multiplicity-adjusted across that sequence. It was also especially vulnerable to un modelled school/class clustering. Together with self-selection into regular use, these limitations preclude a claim that multimedia is educationally superior to AI. Studies of AI-assisted language learning similarly show that adoption, perceived usefulness, enjoyment, and continued use are context- and task-dependent (Huang and Zou, 2024; Liu et al., 2025; Luo and Zou, 2025). The subgroup result should therefore motivate preregistered exposure-stratified research rather than a claim of a stable digital hierarchy.

### 4.4 Robustness and interpretation: what is supported and what remains tentative

The most defensible achievement result is the full-sample proportional-odds association for the textbook evaluation score. Multimedia and AI were not significant in that model, their coefficients did not differ, and the proportional-odds diagnostics were broadly acceptable (Supplementary Table 4). The grade-standardized sensitivity analysis retained the textbook association, while multimedia was small and borderline and AI remained non-significant (Supplementary Table 6). Dimension-level models further showed that the textbook association was present for effectiveness, ease, and enjoyment after Holm adjustment, but not for lower concern or continuance; the composite result was therefore not driven primarily by the concern item (Supplementary Table 8). These analyses improve transparency but do not remove concerns created by locally set examinations, overlapping score bands, uneven grade composition, omitted school/class clustering, and unmeasured respondent type. The cross-sectional design supports description and adjusted association, not causal ordering (Spector, 2019).

### 4.5 Educational meaning of the achievement associations

The positive textbook association has several plausible non-causal explanations. Textbooks are school-sanctioned, aligned with classroom content, and likely to be viewed favorably in a survey distributed by homeroom teachers. Teacher-mediated recruitment may therefore have increased endorsement of the conventional modality. Family support, prior achievement, curricular alignment, and differential access may also influence both evaluations and reported achievement. In contrast, digital and AI tools vary widely in pedagogical quality and the extent to which adults integrate them into accountable learning sequences. Human-AI complementarity research therefore supports guided integration rather than either technological substitution or blanket rejection (Holstein and Aleven, 2022). A practical implication is to evaluate digital resources through concrete instructional roles —such as guided practice, feedback, revision, or extension —rather than through modality labels alone. For younger learners, supervision, task boundaries, age-appropriate content, and opportunities to verify generated responses are likely to be more actionable than asking whether AI is generally better or worse than textbooks. The result should not be translated into a recommendation to replace digital tools with textbooks; it supports only the narrower conclusion that textbook evaluation co-varied with reported achievement in this sample.

### 4.6 Limitations and targeted directions for future work

Several limitations are consequential. First, school and class identifiers were not retained, so clustering from teacher-mediated recruitment could not be modeled and standard errors may be underestimated. This issue is particularly important for the exploratory weekly-exposure subgroup, whose *p* values were not adjusted across the sequence of sensitivity analyses. Second, respondent type was not recorded, preventing parent-vs.-assisted-student comparisons and making parent-only language inappropriate. Third, the convenience sample was concentrated in Grade 5 and county/township locations and is not nationally representative; non-probability recruitment and unavailable partial-response counts further limit population inference (Cornesse et al., 2020; Groves, 2006). Fourth, multimedia and AI ratings were obtained from many non-users or rare users and may capture attitudes rather than experience; the exposure sensitivity analysis was smaller and vulnerable to selection. Fifth, achievement bands overlapped at 90 and 95, varied in width, and came from locally set examinations that were not comparable across grades or schools. Grade standardization addresses only part of this comparability problem because school-level examination difficulty could not be modeled. Sixth, the crossed measurement model was inadmissible, no defensible cross-modality invariance claim can be made, and high internal consistency may reflect halo or method variance. Finally, the cross-sectional design precludes causal or directional inference (Spector, 2019). Future studies should retain school, class, and respondent identifiers; document who completed each item; collect comparable or externally standardized achievement outcomes; distinguish attitudes from evaluations based on verified exposure; randomize recruitment channels where possible; and pretest crossed measurement models before examining structural associations. A longitudinal or experimental design would then be needed to evaluate whether changes in resource use precede changes in achievement rather than merely accompanying them. The administered response options for extracurricular learning time (2–3 h followed by more than 4 h) and daily screen time (1–2 h followed by 3–4 h) leave interval gaps that categorical coding cannot remedy. In addition, parental ability to use English with the child was described but not included as a covariate, leaving potential residual confounding.

## 5 Conclusion

This study compared family-reported use and evaluations of textbooks, multimedia resources, and generative AI tools in elementary English learning. In this teacher-mediated convenience sample, textbooks were used most often and evaluated most favorably, whereas multimedia and AI exposure was substantially lower. The measurement analyses did not validate three separable latent modality predictors, so achievement inference was based on observed scores. Textbook evaluation was positively associated with family-reported achievement band in the full-sample proportional-odds model, whereas multimedia and AI were not. Because recruitment, exposure, measurement, clustering, examination comparability, and cross-sectional design limit inference, the findings describe reported patterns rather than educational effects and should not be generalized to elementary-school families across mainland China.

## Data Availability

De-identified data and the analysis script supporting the reported results are available from the corresponding author without undue reservation, subject to safeguards required for research involving minors and applicable institutional review.
