# Supplementary material for: Textbook, multimedia, and generative AI in elementary English learning: usage patterns and family-reported evaluations
Source: Front Psychol. 2026 Oct 7;17:1870251. doi: 10.3389/fpsyg.2026.1870251 (PMC13685599; doi:10.3389/fpsyg.2026.1870251)
Supplement: Supplementary file 1 [file Supplementary_File_1.docx]

## Supplementary Material

**Table S1. Item descriptive statistics and internal consistency**

Panel A. Item-level descriptive statistics

| Item | n | Mean | SD | Median | Min | Max | Skewness | Kurtosis |
| --- | --- | --- | --- | --- | --- | --- | --- | --- |
| Eff_TB | 987 | 3.811 | 1.074 | 4 | 1 | 5 | -0.669 | -0.008 |
| Eff_MM | 987 | 3.183 | 1.235 | 3 | 1 | 5 | -0.312 | -0.668 |
| Eff_AI | 987 | 3.107 | 1.218 | 3 | 1 | 5 | -0.216 | -0.662 |
| Ease_TB | 987 | 3.427 | 1.218 | 3 | 1 | 5 | -0.181 | -1.069 |
| Ease_MM | 987 | 2.990 | 1.246 | 3 | 1 | 5 | 0.000 | -0.892 |
| Ease_AI | 987 | 3.019 | 1.257 | 3 | 1 | 5 | -0.045 | -0.931 |
| Enjoy_TB | 987 | 3.483 | 1.043 | 3 | 1 | 5 | -0.280 | -0.109 |
| Enjoy_MM | 987 | 3.392 | 1.194 | 3 | 1 | 5 | -0.443 | -0.474 |
| Enjoy_AI | 987 | 3.210 | 1.185 | 3 | 1 | 5 | -0.286 | -0.548 |
| Conc_TB | 987 | 3.925 | 1.130 | 4 | 1 | 5 | -0.865 | 0.008 |
| Conc_MM | 987 | 2.968 | 1.162 | 3 | 1 | 5 | -0.057 | -0.622 |
| Conc_AI | 987 | 2.911 | 1.189 | 3 | 1 | 5 | -0.030 | -0.690 |
| Cont_TB | 987 | 4.070 | 1.043 | 4 | 1 | 5 | -0.971 | 0.367 |
| Cont_MM | 987 | 3.244 | 1.200 | 3 | 1 | 5 | -0.294 | -0.612 |
| Cont_AI | 987 | 3.155 | 1.209 | 3 | 1 | 5 | -0.226 | -0.644 |

Panel B. Internal consistency by modality

| Modality | Cronbach’s α | Standardized α | Mean inter-item r |
| --- | --- | --- | --- |
| Textbook | 0.869 | 0.871 | 0.574 |
| Multimedia | 0.908 | 0.908 | 0.664 |
| AI | 0.905 | 0.905 | 0.657 |

**Note.** Items were rated from 1 to 5, with higher scores indicating more favourable evaluations. Eff = perceived effectiveness; Ease = ease of use; Enjoy = enjoyment; Conc = concern, oriented so that higher scores indicate lower concern; Cont = continuance intention. TB = textbook; MM = multimedia; AI = AI-based tools. Internal consistency was computed for the five evaluation items within each modality.

**Table S2. Exploratory factor analysis diagnostics and loading patterns**

Panel A. Factorability diagnostics and parallel analysis

| Diagnostic | Value |
| --- | --- |
| EFA sample size | 493 |
| Number of items | 15 |
| KMO | 0.818 |
| Bartlett χ² | 7334.595 |
| Bartlett df | 105 |
| Bartlett p | <.001 |
| Parallel analysis suggested factors | 4 |
| Minimum eigenvalue after smoothing check | 0.050 |

Panel B. Three-factor loading pattern

| Item | PA1 | PA3 | PA2 |
| --- | --- | --- | --- |
| Eff_TB |  |  | 0.846 |
| Eff_MM |  | 0.929 |  |
| Eff_AI | 0.868 |  |  |
| Ease_TB |  |  | 0.738 |
| Ease_MM |  | 0.769 |  |
| Ease_AI | 0.815 |  |  |
| Enjoy_TB |  |  | 0.789 |
| Enjoy_MM |  | 0.936 |  |
| Enjoy_AI | 0.817 |  |  |
| Conc_TB |  |  | 0.737 |
| Conc_MM |  | 0.636 |  |
| Conc_AI | 0.802 |  |  |
| Cont_TB |  |  | 0.835 |
| Cont_MM |  | 0.799 |  |
| Cont_AI | 0.889 |  |  |

Panel C. Four-factor loading pattern

| Item | PA1 | PA3 | PA2 | PA4 |
| --- | --- | --- | --- | --- |
| Eff_TB |  |  | 0.843 |  |
| Eff_MM |  | 0.884 |  |  |
| Eff_AI | 0.828 |  |  |  |
| Ease_TB |  |  | 0.745 |  |
| Ease_MM |  | 0.793 |  |  |
| Ease_AI | 0.859 |  |  |  |
| Enjoy_TB |  |  | 0.786 |  |
| Enjoy_MM |  | 0.906 |  |  |
| Enjoy_AI | 0.790 |  |  |  |
| Conc_TB |  |  | 0.743 |  |
| Conc_MM |  | 0.645 |  | 0.397 |
| Conc_AI | 0.769 |  |  | 0.377 |
| Cont_TB |  |  | 0.839 |  |
| Cont_MM |  | 0.791 |  |  |
| Cont_AI | 0.817 |  |  |  |

Panel D. Variance accounted

| Solution | Factor | SS loadings | Proportion variance | Cumulative variance |
| --- | --- | --- | --- | --- |
| Three-factor | PA1 | 3.711 | 0.247 | 0.247 |
| Three-factor | PA3 | 3.513 | 0.234 | 0.482 |
| Three-factor | PA2 | 3.192 | 0.213 | 0.694 |
| Four-factor | PA1 | 3.654 | 0.244 | 0.244 |
| Four-factor | PA3 | 3.586 | 0.239 | 0.483 |
| Four-factor | PA2 | 3.266 | 0.218 | 0.700 |
| Four-factor | PA4 | 0.694 | 0.046 | 0.747 |

Note. Exploratory analyses used the EFA subsample and a polychoric correlation matrix. Extraction used principal-axis factoring with oblimin rotation. Loadings below |0.30| are omitted. In both the three- and four-factor solutions, the main loading pattern was modality-based. The fourth factor accounted for only 4.6% of variance and consisted mainly of secondary concern-item loadings, rather than a coherent substantive factor; it was therefore not carried forward as a confirmatory candidate. A five-factor solution produced an ultra-Heywood warning and was not retained as a substantive model.

**Table S3. Standardized CFA loadings and latent factor correlations for the original three-modality model**

Panel A. Standardized factor loadings

| Factor | Item | Estimate | SE | z | p | Std. loading |
| --- | --- | --- | --- | --- | --- | --- |
| Textbook | Eff_TB | 0.832 | 0.018 | 46.714 | <.001 | 0.832 |
| Textbook | Ease_TB | 0.785 | 0.022 | 35.336 | <.001 | 0.785 |
| Textbook | Enjoy_TB | 0.821 | 0.018 | 44.828 | <.001 | 0.821 |
| Textbook | Conc_TB | 0.873 | 0.015 | 58.219 | <.001 | 0.873 |
| Textbook | Cont_TB | 0.889 | 0.016 | 56.967 | <.001 | 0.889 |
| Multimedia | Eff_MM | 0.849 | 0.013 | 67.319 | <.001 | 0.849 |
| Multimedia | Ease_MM | 0.830 | 0.015 | 55.453 | <.001 | 0.830 |
| Multimedia | Enjoy_MM | 0.838 | 0.014 | 58.478 | <.001 | 0.838 |
| Multimedia | Conc_MM | 0.841 | 0.014 | 60.301 | <.001 | 0.841 |
| Multimedia | Cont_MM | 0.891 | 0.011 | 79.536 | <.001 | 0.891 |
| AI | Eff_AI | 0.830 | 0.014 | 58.297 | <.001 | 0.830 |
| AI | Ease_AI | 0.837 | 0.014 | 60.327 | <.001 | 0.837 |
| AI | Enjoy_AI | 0.849 | 0.013 | 67.486 | <.001 | 0.849 |
| AI | Conc_AI | 0.869 | 0.012 | 73.365 | <.001 | 0.869 |
| AI | Cont_AI | 0.859 | 0.013 | 66.322 | <.001 | 0.859 |

Panel B. Latent factor correlations

| Factor 1 | Factor 2 | Correlation | SE | z | p |
| --- | --- | --- | --- | --- | --- |
| Textbook | Multimedia | 0.403 | 0.038 | 10.754 | <.001 |
| Textbook | AI | 0.326 | 0.039 | 8.454 | <.001 |
| Multimedia | AI | 0.847 | 0.019 | 45.420 | <.001 |

**Note.** The original three-modality CFA was estimated in the CFA subsample using WLSMV with ordered indicators. Its absolute fit was poor (RMSEA = .151). The Multimedia-AI latent correlation of .847 indicates limited discriminability. These estimates are retained as measurement diagnostics and are not used as validated latent predictors.

**Table S4. Primary proportional-odds model for family-reported English achievement band**

Panel A. Full cumulative-logit coefficient table

| **Predictor** | **b (SE)** | **OR; 95% CI for b** | **p** |
| --- | --- | --- | --- |
| Textbook score | .343 (.082) | 1.409 [.182, .503] | <.001 |
| Multimedia score | .147 (.085) | 1.158 [-.020, .313] | .085 |
| AI score | -.0197 (.081) | .981 [-.179, .139] | .808 |
| Grade 2 | .157 (.334) | 1.170 [-.498, .812] | .638 |
| Grade 3 | .443 (.278) | 1.558 [-.102, .989] | .111 |
| Grade 4 | 2.019 (.283) | 7.534 [1.465, 2.573] | <.001 |
| Grade 5 | 1.740 (.250) | 5.695 [1.250, 2.230] | <.001 |
| Grade 6 | 2.075 (.486) | 7.967 [1.123, 3.027] | <.001 |
| Female | .141 (.122) | 1.152 [-.098, .380] | .247 |
| County/township | -.083 (.178) | .920 [-.432, .267] | .642 |
| Rural | .281 (.673) | 1.325 [-1.038, 1.600] | .676 |
| Senior secondary vs junior secondary or below | .451 (.184) | 1.569 [.089, .812] | .015 |
| College vs junior secondary or below | .866 (.197) | 2.377 [.480, 1.251] | <.001 |
| Undergraduate vs junior secondary or below | 1.242 (.201) | 3.463 [.848, 1.637] | <.001 |
| Postgraduate vs junior secondary or below | 1.707 (.348) | 5.511 [1.024, 2.389] | <.001 |
| English-learning interest | .801 (.077) | 2.229 [.651, .952] | <.001 |
| About 1 hour vs almost none | .211 (.163) | 1.235 [-.108, .529] | .195 |
| 1-2 hours vs almost none | .040 (.175) | 1.041 [-.303, .383] | .819 |
| 2-3 hours vs almost none | .159 (.221) | 1.172 [-.274, .592] | .471 |
| More than 4 hours vs almost none | .583 (.278) | 1.791 [.038, 1.127] | .036 |
| 1-2 hours vs <1 hour | -.074 (.131) | .929 [-.330, .182] | .571 |
| 3-4 hours vs <1 hour | -.130 (.315) | .878 [-.748, .488] | .680 |
| >=5 hours vs <1 hour | .158 (.461) | 1.172 [-.745, 1.061] | .731 |
| English books/picture books | .211 (.134) | 1.235 [-.052, .473] | .115 |
| Learning app/platform | .192 (.145) | 1.212 [-.092, .476] | .186 |
| Quiet study place | .176 (.134) | 1.192 [-.086, .438] | .188 |

Panel B. Nominal-effect likelihood-ratio tests of the proportional-odds assumption

| **Term** | **df** | **LR chi-square** | **p** |
| --- | --- | --- | --- |
| Textbook score | 3 | 3.369 | .338 |
| Multimedia score | 3 | 2.596 | .458 |
| AI score | 3 | 1.277 | .735 |
| Grade | 15 | 20.256 | .162 |
| Gender | 3 | 6.489 | .090 |
| Region | 6 | 12.553 | .051 |
| Parental education | 12 | 16.733 | .160 |
| English-learning interest | 3 | 1.995 | .573 |
| Extracurricular-learning time | 12 | 16.890 | .154 |
| Screen time | 9 | 8.624 | .473 |
| Books | 3 | 5.335 | .149 |
| Learning app/platform | 3 | 3.756 | .289 |
| Quiet study place | 3 | .306 | .959 |

Note. N = 987. Modality scores are means of the five evaluation items. Grade, gender, region, parental education, extracurricular-learning time, and screen time were entered categorically; English-learning interest and binary home-resource indicators were entered as recorded. Confidence intervals are Wald intervals for cumulative-logit coefficients, and odds ratios were calculated from unrounded coefficients. The nominal-effect tests did not detect a proportional-odds violation at p < .05, although region was borderline (p = .051). School/class clustering could not be estimated because identifiers were unavailable. Reference categories were Grade 1, male, urban, junior secondary or below, almost no extracurricular English-learning time, and less than 1 hour of daily screen time.

Table S5. Usage exposure and weekly-digital-user sensitivity analysis

Panel A. Usage exposure distribution

| **Modality** | **Never** | **Never or rarely** | **At least weekly** |
| --- | --- | --- | --- |
| Textbook | 72 (7.3%) | 177 (17.9%) | 810 (82.1%) |
| Multimedia | 378 (38.3%) | 579 (58.7%) | 408 (41.3%) |
| AI | 300 (30.4%) | 532 (53.9%) | 455 (46.1%) |

Panel B. Proportional-odds model among respondents using both multimedia and AI at least weekly (n = 293)

| **Term** | **b** | **95% CI** | **p** |
| --- | --- | --- | --- |
| Textbook score | .347 | [-.041, .736] | .080 |
| Multimedia score | .654 | [.173, 1.135] | .008 |
| AI score | -.273 | [-.729, .183] | .241 |
| Multimedia - AI coefficient | .926 | [.066, 1.790] | .035 |

Note. At least weekly corresponds to Q16 category 3 or higher. Panel A reports exposure distributions; Panel B reports the subgroup model. The exposed subgroup represented 29.7% of the analytic sample. Because restriction conditions on self-selected exposure, the subgroup followed a non-significant full-sample contrast among several sensitivity analyses, its p values were not adjusted across that sequence, and school/class clustering was unavailable, Panel B is exploratory.

Table S6. Grade-standardized achievement sensitivity analysis

| **Predictor** | **b** | **95% CI** | **p** |
| --- | --- | --- | --- |
| Textbook score | .142 | [.071, .213] | <.001 |
| Multimedia score | .074 | [.002, .147] | .045 |
| AI score | -.021 | [-.090, .049] | .558 |

**Note.** Achievement-band codes were standardized within grade before fitting the controlled linear sensitivity model. School-level standardization was not possible because school identifiers were unavailable.

Table S7. Full wording and response anchors for the modality items

| **Item** | **Prompt** | **Response anchors** |
| --- | --- | --- |
| Q16 Usage | In the past two weeks, how frequently has your child used the following ways to learn English? | 1 Never; 2 Rarely (a few times/month); 3 Sometimes (1-2 times/week); 4 Often (3-4 times/week); 5 Almost daily |
| Q17 Effectiveness | In your opinion, how effective is each way of learning English? | 1 Very poor; 2 Poor; 3 Neutral; 4 Good; 5 Very good |
| Q18 Ease of use | When using these ways to learn, how difficult is it for your child to operate or use them? | 1 Very difficult; 2 Somewhat difficult; 3 Moderate; 4 Relatively easy; 5 Very easy |
| Q19 Enjoyment | When using these ways to learn, how interesting is each one for your child? | 1 Very boring; 2 Boring; 3 Neutral; 4 Interesting; 5 Very interesting |
| Q20 Concern | For these learning ways, how concerned are you about potential negative effects? | 1 Very worried; 2 Worried; 3 Somewhat worried; 4 Not very worried; 5 Not worried at all |
| Q21 Continuance | In the future, would you still be willing to let your child continue using these ways to learn? | 1 Strongly unwilling; 2 Unwilling; 3 Neutral; 4 Willing; 5 Strongly willing |

**Note.** Each prompt was repeated for Textbook/traditional materials, Multimedia courses/apps, and AI tools. The wording is an English translation of the administered Chinese questionnaire.

Table S8. Dimension-level proportional-odds sensitivity analyses

| **Dimension** | **Modality** | **b** | **SE** | **95% CI** | **OR** | **p** | **p_Holm** |
| --- | --- | --- | --- | --- | --- | --- | --- |
| Effectiveness | Textbook | .208 | .062 | [.087, .329] | 1.231 | .001 | .003 |
| Effectiveness | Multimedia | .133 | .062 | [.011, .255] | 1.142 | .033 | .132 |
| Effectiveness | AI | -.040 | .062 | [-.161, .080] | .961 | .513 | 1.000 |
| Ease | Textbook | .350 | .062 | [.229, .471] | 1.419 | <.001 | <.001 |
| Ease | Multimedia | .171 | .070 | [.034, .309] | 1.187 | .014 | .072 |
| Ease | AI | -.031 | .065 | [-.159, .096] | .969 | .631 | 1.000 |
| Enjoyment | Textbook | .213 | .068 | [.080, .345] | 1.237 | .002 | .005 |
| Enjoyment | Multimedia | .036 | .064 | [-.090, .162] | 1.037 | .580 | 1.000 |
| Enjoyment | AI | .015 | .063 | [-.108, .138] | 1.015 | .810 | 1.000 |
| Lower concern | Textbook | .104 | .060 | [-.013, .222] | 1.110 | .082 | .145 |
| Lower concern | Multimedia | .050 | .077 | [-.101, .202] | 1.052 | .517 | 1.000 |
| Lower concern | AI | .020 | .072 | [-.122, .162] | 1.020 | .781 | 1.000 |
| Continuance | Textbook | .115 | .064 | [-.010, .241] | 1.122 | .072 | .145 |
| Continuance | Multimedia | .086 | .072 | [-.054, .226] | 1.090 | .229 | .686 |
| Continuance | AI | .047 | .068 | [-.086, .180] | 1.048 | .491 | 1.000 |

Note. N = 987. Five separate proportional-odds models were fitted. Each model simultaneously entered the textbook, multimedia, and AI ratings for one evaluation dimension and used the same measured background controls as the primary model. For concern, higher values indicate lower concern. p_Holm adjusts the five dimension-specific tests within each modality. Confidence intervals are unadjusted 95% Wald intervals; adjusted p values are used for multiplicity-aware inference. School/class clustering could not be estimated because identifiers were unavailable.
